# Efficacy and safety evaluation of hyperbaric oxygen therapy for patients with ulcerative colitis: A protocol of systematic review and meta-analysis

**DOI:** 10.1097/MD.0000000000023966

**Published:** 2021-01-08

**Authors:** Wei Wang, Ying He, Dou Wen, Shangshang Jiang, Xiaodong Zhao

**Affiliations:** a Dongzhimen Hospital, Beijing University of Chinese Medicine; b Hospital of Chengdu University of Traditional Chinese Medicine, Chengdu, Sichuan, P.R. China.

**Keywords:** hyperbaric oxygen therapy, protocol, systematic review, ulcerative colitis

## Abstract

**Background:**

Ulcerative colitis (UC) belongs to chronic colitis whose etiology and pathogenesis still have remained unclear. Hyperbaric oxygen therapy (HBOT) has been demonstrated to be effective for UC therapy. Still, evidence of its efficacy and safety is inconclusive. The purpose of the protocol is to evaluate the efficacy and safety of HBOT in UC therapy.

**Methods:**

This systematic review will retrieve studies that meet the requirements in Embase, MEDLINE, PubMed, Web of Science, Cochrane Library Central Register of Controlled Trials, the Chinese Biomedical Literature Database (CBM), China national knowledge infrastructure database (CNKI), Wei Pu database, Wan fang database, SinoMed, Google scholar, and Baidu Scholar from their inception to November 2020. Two authors are to be independent in their article selection, data collection, and research quality assessments. The primary outcome is the clinical effectiveness. And the secondary outcomes will include 4 criteria. RevMan 5.3 software will be utilized for analysis of the data.

**Results:**

The results of this study are to be submitted via a peer-reviewed journal.

**Conclusions:**

The study is to assess the effectiveness and safety of HBOT for UC and provide valid and reliable evidence regarding HBOT for UC.

**INPLASY registration number:**

INPLASY2020100118.

## 1 Introduction

Ulcerative colitis (UC) is chronic colitis affecting the bowel whose pathogenic mechanism is multifactored. The pathogenic mechanism of UC involves genetic, immune, and environmental factors.^[1]^ Symptoms of UC usually present with bloody mucopurulent stool, diarrhea, and bellyache, and they seriously impair the level of health for patients.^[2]^ In addition, diagnosis of UC mainly relies on endoscopy and clinical symptoms.^[3]^ It has been reported that the occurrence rate of UC is increasing globally.^[4]^ Furthermore, the incidence of UC is increasing worldwide over the past few years, affecting mostly young individuals.^[5,6]^ And UC is becoming one of the clinically refractory diseases.

Nowadays, drug treatment and surgery have become the major treatment modalities for UC.^[7]^ More specifically, the effective drug treatments for UC are 5-aminosalicylate compounds, corticosteroids, and immunosuppressive agents. However, failure of standard drug treatments for UC often leads to colon removed.^[8]^ In a word, poor efficacy and side effects of drug treatment and surgery would hinder the health for some UC patients. Thus, there is an urgent need to explore novel treatment options against UC.

Hyperbaric oxygen therapy (HBOT) is a promising medical technology in which people breath 100% oxygen under elevated atmospheric pressure.^[9]^ HBOT not only improves plasma and tissue oxygen content but also improves the oxygen levels of blood reaching inflamed bowel.^[10]^ Indeed, several clinical trials have suggested that HBOT is effective for UC therapy,^[11–13]^ and the mechanism of action of HBOT for UC could be associated with reduction of pro-inflammatory cytokines and chemokines which are responsible for the metabolic stress created during active inflammation.^[10]^ However, there are no previously published systematic review and meta-analyses on assessing the effectiveness and safety of HBOT for UC, and evidence of its efficacy and safety is inconclusive. Therefore, the article intends to adopt the systematic evaluation and meta-analysis to assess the clinical effectiveness and safety of HBOT for UC, so as to offer the scientific evidence of HBOT for UC.

## 2 Methods

### 2.1 Study registration

We registered the protocol with the International Platform of Registered Systematic Review and Meta-Analysis Protocols (INPLASY) on 31 October 2020. Its accession number is INPLASY2020100118 (DOI is 10.37766/inplasy2020.10.0118, https://inplasy.com/inplasy-2020-10-0118/).

### 2.2 Inclusion criteria

#### 2.2.1 Selection of researches

Randomized controlled trials (RCTs) that investigated the effectiveness and safety of HBOT for UC will be selected for inclusion. Non-randomized controlled trials, literature reviews, case reports, studies with animal experiments, specialist experience, and repeated documents will not be collected.

#### 2.2.2 Selection of participants

Participants who are clinically met the diagnostic criteria of UC are to be enrolled, in despite of sex, age, ethnicity, the extent of illness, economic status, or educational level. Meanwhile, some special UC participants who were pregnant or breastfeeding mothers or with cardiac, hepatic or lung diseases will be excluded.

#### 2.2.3 Selection of intervention

On the one hand, the intervention measures of the treatment group were individual HBOT or in collaboration with conventional therapy according to modern guidelines. On the other hand, the intervention measures of the control group included no HBOT therapy, placebo and conventional treatment according to modern guidelines.

#### 2.2.4 Settings of outcome measures

We will mainly observe the overall effectiveness to obtain the primary outcomes. And the secondary outcomes will contain 4 items: clinical response, endoscopic remission, inflammatory markers levels, and adverse events.

### 2.3 Search strategy

We are to systematically search medical subject headings and keywords associated with HBOT in the treatment of UC based on the following databases until November 2020: Embase, MEDLINE, PubMed, Web of Science, Cochrane Library Central Register of Controlled Trials, the Chinese Biomedical Literature Database (CBM), China national knowledge infrastructure database (CNKI), Wei Pu database, Wan fang database, and SinoMed. Meanwhile, we will also search the relevant literatures in Google scholar, Baidu Scholar. Table 1 shows the search strategy of PubMed. Likewise, similar strategies slightly modified were used for the other databases.

**Table 1:** Search strategy in PubMed.

Number	Entry terms
#1	“Colitis, Ulcerative” [MeSH Terms] or “Colitis, Ulcerative” [Title/Abstract] or “Idiopathic Proctocolitis” [Title/Abstract] or “Ulcerative Colitis” [Title/Abstract] or “Colitis Gravis” [Title/Abstract] or “ Inflammatory Bowel Disease, Ulcerative Colitis Type” [Title/Abstract]
#2	“Hyperbaric Oxygenation” [MeSH Terms] or “Hyperbaric Oxygenation” [Title/Abstract] or “Hyperbaric Oxygenations” [Title/Abstract] or “Oxygenations, Hyperbaric” [Title/Abstract] or “Hyperbaric Oxygen Therapy” [Title/Abstract] or “Hyperbaric Oxygen Therapies” [Title/Abstract] or “Oxygen Therapies, Hyperbaric” [Title/Abstract] or “Oxygen Therapy, Hyperbaric” [Title/Abstract] or “Therapies, Hyperbaric Oxygen” [Title/Abstract] or “Therapy, Hyperbaric Oxygen” [Title/Abstract] or “Oxygenation, Hyperbaric” [Title/Abstract]
#3	“Randomized controlled trial” [Publication Type] or “Randomized controlled trial” [Title/Abstract]
#4	#1 and #2 and #3

### 2.4 Data obtainment and analysis

#### 2.4.1 Collection of articles

**Figure 1:**
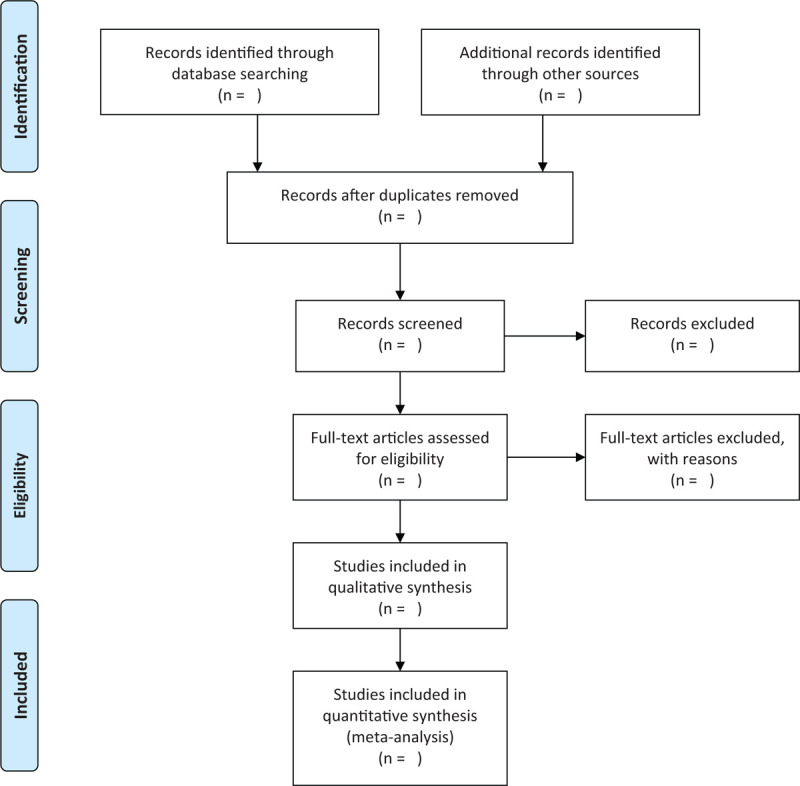
Flow diagram of the study selection process.

Firstly, according to the search strategies, the preliminary articles are to be searched and imported to EndnoteX9 tool. And then, the EndnoteX9 tool will be used to remove the duplicates. After this, 2 authors will review the titles and abstracts of the studies independently to narrow down the articles. Next, the eligible articles will be read for full-text assessment to be further screened by 2 authors. In case of disagreements, we will consult the third author and resolve them by discussions. This selection process is demonstrated by a flow chart as shown in Fig. 1.

#### 2.4.2 Data refinement

Two authors are to independently refine the data from the articles which met the eligibility, containing routine information of articles, study approaches, all participant information, the content of control and the intervention, outcome indexes, and adverse effects. Should data be missing, inaccurate or ambiguous, we will resolve it via contacting the corresponding author in obtained articles, or consulting the third party or conducting internal discussions.

#### 2.4.3 Evaluation of risk of bias

Two authors will independently assess the risk of bias on the basis of the Cochrane risk-of-bias tool for each study included. And they will determine the bias based on these aspects: random sequence generation, concealed allocation, implementation of blind method of the participants and subjects, implementation of blind method of the research results, data integrity, reporting with selectivity, and other aspects. In the end, each study will be evaluated into 3 conditions, “Low risk,” “High risk,” and “Unclear risk.”^[14]^ In addition, we will resolve any inconsistencies through contacting the corresponding author in obtained literature, consulting the third party, or conducting internal discussions.

#### 2.4.4 Determination of treatment efficacy

For continuous data, we are to apply the mean difference with 95% confidence intervals to determine the treatment efficacy. For dichotomous data, we are to apply relative risk with 95% confidence intervals for analysis.

#### 2.4.5 Handling missing data

Where data are missing, we need attempt to communicate with the first author or correspondent author by e-mail or phone to acquire the complete data. If the contact fails, we will start our analysis according to the existing data.^[15]^ Additionally, the probable effect of incomplete data will also be included in the discussion.

#### 2.4.6 Determination of heterogeneity

Heterogeneity will be determined with the *I*^2^ statistic and chi-squared test. There is substantial heterogeneity among the trials when *I*^2^ ≥ 50%, but low or no heterogeneity while *I*^2^ < 50%. Moreover, we will conduct subgroup analysis or sensitivity analysis to check the possible reasons when heterogeneity is substantial.

#### 2.4.7 Analysis of reporting bias

We will apply funnel plots and Egger test to detect reporting bias if at least 10 RCTs are available to us.^[16]^

#### 2.4.8 Data synthesis

Related analyses will be performed in RevMan 5.3 software (The Cochrane Collaboration, Oxford, England). If heterogeneity is minor (*I*^2^ < 50%), we will apply the fixed-effect approach for meta-analysis. However, if heterogeneity is substantial *(I*^2^ ≥ 50%), we will apply the random-effect approach for meta-analysis. On the other hand, we will carry out the narrative analysis if there is significant heterogeneity or inability to judge the source of it.

#### 2.4.9 Subgroup analysis

If necessary, we need carry out subgroup analysis to deal with heterogeneity due to interventions, participant information, and outcome indexes.

#### 2.4.10 Sensitivity analysis

We are to conduct sensitivity analysis to assess the stability of the conclusions by eliminating the inferior methodological quality studies.

#### 2.4.11 Determination of evidence quality

We will use the Grading of Recommendations Assessment, Development, and Evaluation (GRADE) working group approach to determine the evidence quality for all outcomes. The quality of evidence quality will be classified into as high, moderate, low, or very low.

### 2.5 Ethics and dissemination

The study does not need ethics approval for the reason that primary personal data will not be collected. In addition, the results of this study are to be submitted via a peer-reviewed journal.

## 3 Discussion

UC is a refractory and immune-related intestinal lesion with repeated attack and unknown etiology, which substantially diminishes the quality of life of patients.^[17]^ HBOT is a commonly primary or adjunctive medical treatment^[18]^ and more and more clinical studies suggest it is potentially beneficial to therapy for UC.^[11–13,19,20]^ Nevertheless, there are no systemic reviews published regarding the effectiveness and safety of HBOT for UC. Consequently, we will perform the study to determine the effectiveness and safety of HBOT for UC. Similarly, it is our hope that this study will supply further evidence to guide clinical practice for UC. Additionally, it is important to notice that this study still has several potential limitations. First, the quality of different study could lead to the substantial heterogeneity. Second, the different modalities of HBOT and the different conditions of patients with UC may also cause clinical heterogeneity.

## Author contributions

**Data curation:** Wei Wang, Ying He, Shangshang Jiang.

**Formal analysis:** Wei Wang, Ying He, Shangshang Jiang.

**Methodology:** Wei Wang, Ying He, Dou Wen.

**Project administration:** Shangshang Jiang, Xiaodong Zhao.

**Resources:** Dou Wen, Xiaodong Zhao.

**Software:** Wei Wang, Ying He, Dou Wen.

**Visualization:** Wei Wang, Ying He, Xiaodong Zhao.

**Writing – original draft:** Wei Wang, Ying He.

**Writing – review & editing:** Shangshang Jiang, Xiaodong Zhao.
